# Supplementary material for: Report on Vincristine-Producing Endophytic Fungus Nigrospora zimmermanii from Leaves of Catharanthus roseus
Source: Metabolites. 2022 Nov 15;12(11):1119. doi: 10.3390/metabo12111119 (PMC9697034; doi:10.3390/metabo12111119)
Supplement: Supplementary file 1 [file metabolites-12-01119-s001.zip › metabolites-2000690-supplementary.pdf]

Table S1. GenBank accessions of the sequence analysis.

Description	Per. ident	Acc. Len	Accession
Nigrospora zimmermanii CBS 290.62 ITS region; from TYPE material	99.24	549	NR_153485.1
Nigrospora pyriformis CGMCC 3.18122 ITS region; from TYPE material	97.52	540	NR_153469.1
Nigrospora vesicularifera CGMCC 3.19333 ITS region; from TYPE material	97.14	528	NR_165927.1
Nigrospora sacchari-officinarum CGMCC 3.19335 ITS region; from TYPE material	96.58	536	NR_165926.1
Nigrospora hainanensis CGMCC 3.18129 ITS region; from TYPE material	96.59	533	NR_153480.1
Nigrospora aurantiaca CGMCC 3.18130 ITS region; from TYPE material	96.76	526	NR_153477.1
Nigrospora camelliae-sinensis CGMCC 3.18125 ITS region; from TYPE material	97.46	511	NR_153473.1
Nigrospora vesicularis CGMCC 3.18128 ITS region; from TYPE material	96.38	526	NR_153479.1
Nigrospora guilinensis CGMCC 3.18124 ITS region; from TYPE material	96.14	516	NR_153472.1
Nigrospora bambusae CGMCC 3.18327 ITS region; from TYPE material	96.23	482	NR_153484.1

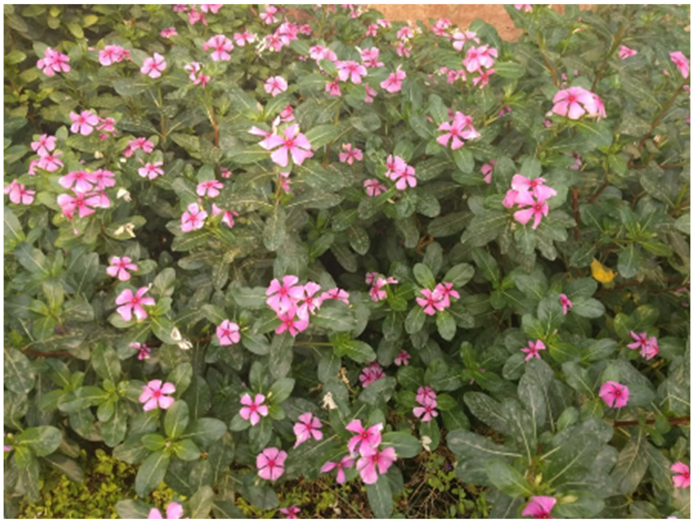

Figure S1. *Catharanthus roseus (L.) G. Don* habit.

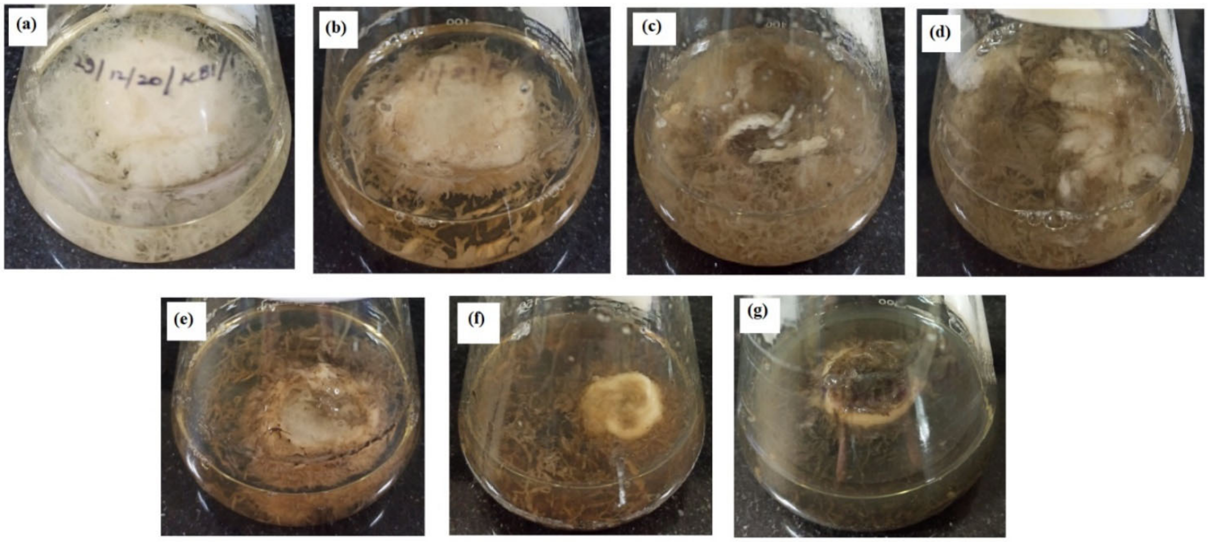

Figure S2. Eight-day suspension culture in PDB media; (a) Parent fungus [Generation 1]; (b) Generation 2; (c) Generation 3; (d) Generation 4; (e) Generation 5; (f) Generation 6; (g) Generation7.

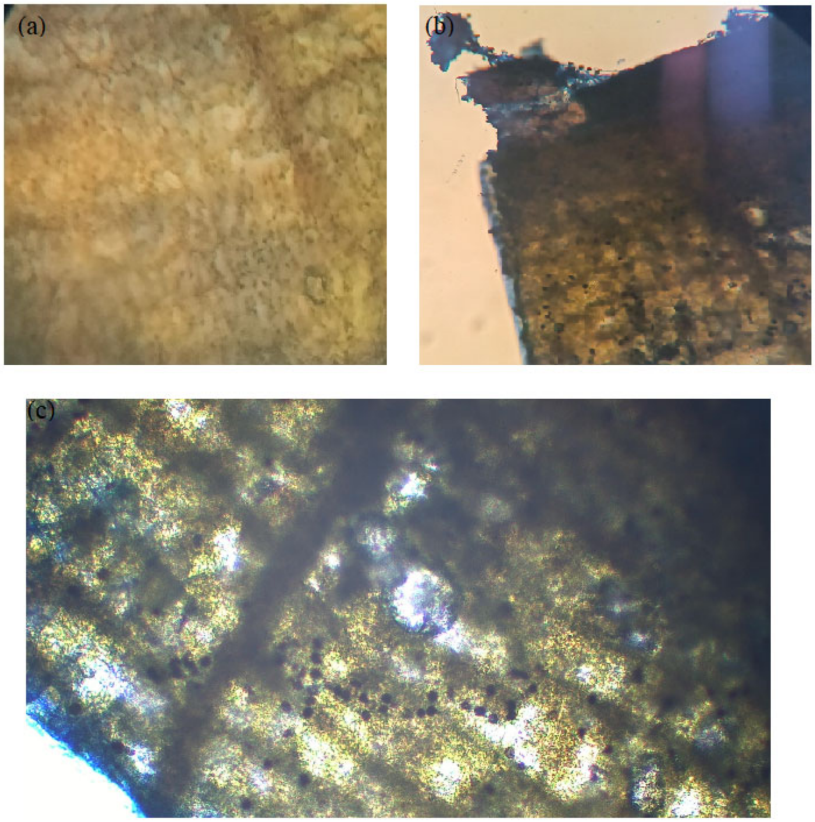

Figure S3. Colonization study: (a) Control leaf sample before infection (no endophytic spores or hypha visible under light microscope); (b) Mycelia oozing out of the leaf surface visible under the microscope at 100x magnification; (c) Spores (blue) visible under the microscope at 400x magnification inside the leaf-cutting of *Catharanthus roseus* stained with lactophenol cotton blue.

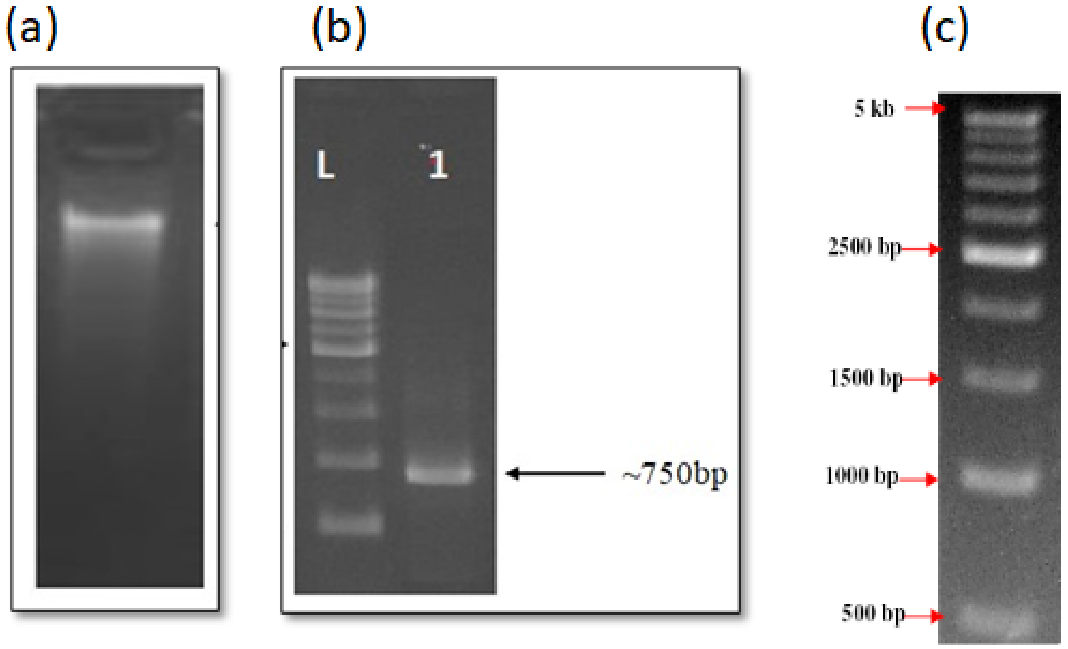

Figure S4. (**a**) gDNA isolated from fungus *Nigrospora zimmermanii* (**b**) Band for PCR product of ITS region at ~750bp (**c**) 500 bp ladder contains 10 DNA fragments of size 500 bp, 1000 bp, 1500 bp, 2000 bp, 2500 bp, 3000 bp, 3500 bp, 4000 bp, 4500 bp and 5000 bp.

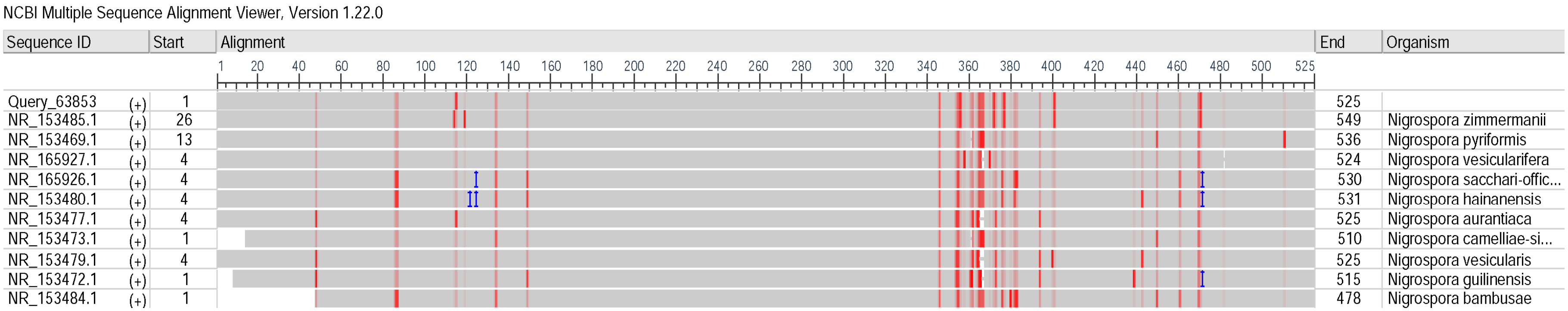

Figure S5. Sequences producing significant alignments data, generated by NCBI Multiple Sequence Alignment Viewer, Version 1.22.0.

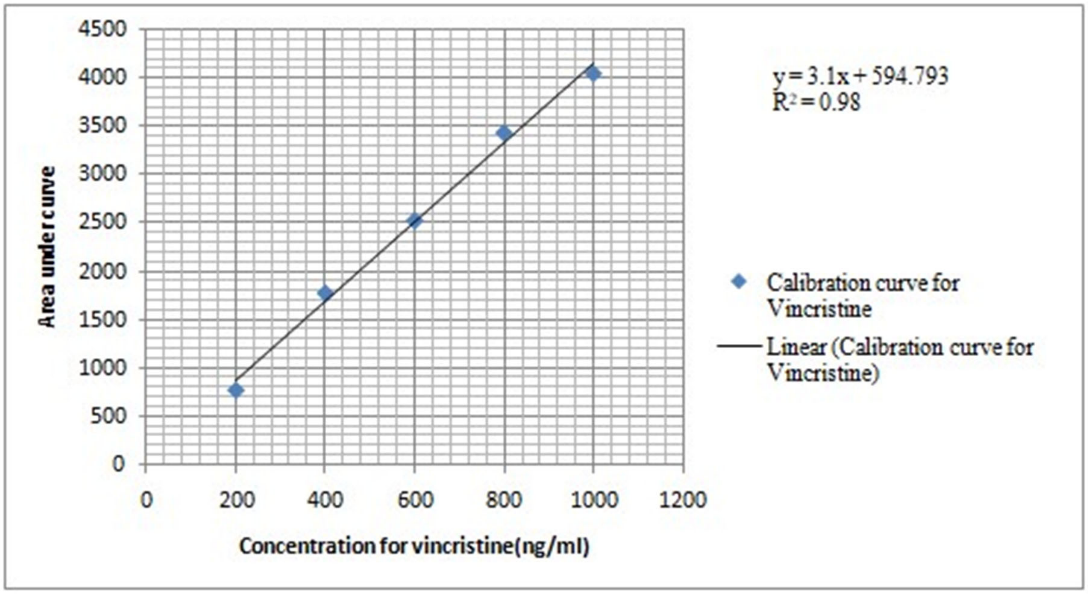

Figure S6. Calibration curve for vincristine standard.

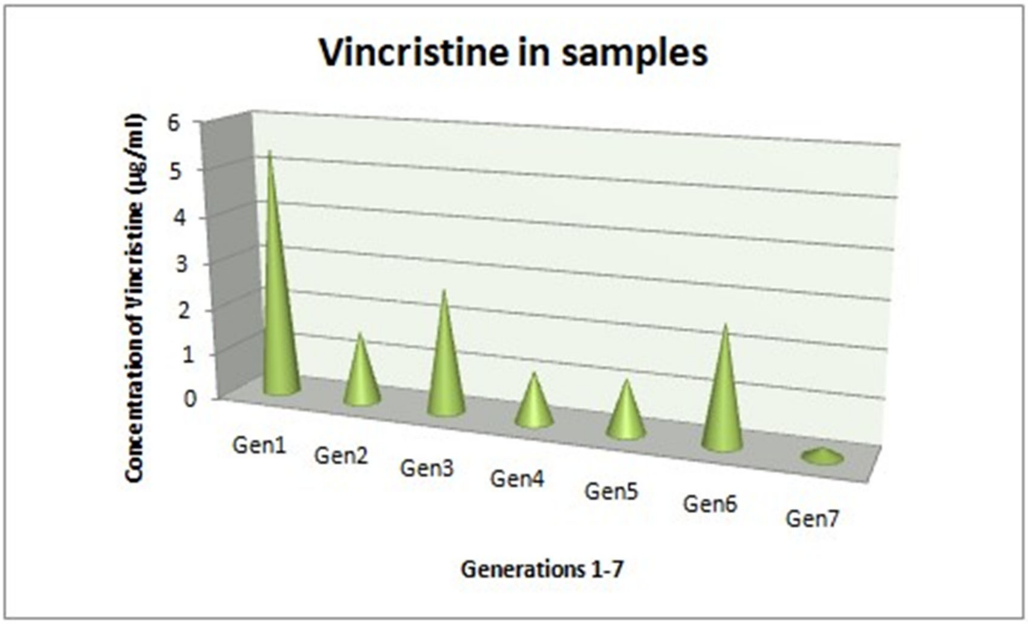

Figure S7. Graphical representation of the quantity of vincristine in samples from Generation 1 to 7.
